# Quality of life and mortality from a nephrologist's view: a prospective observational study

**DOI:** 10.1186/1471-2369-10-39

**Published:** 2009-11-24

**Authors:** Seung Seok Han, Ki Woong Kim, Ki Young Na, Dong-Wan Chae, Yon Su Kim, Suhnggwon Kim, Ho Jun Chin

**Affiliations:** 1 Department of Internal Medicine, Seoul National University College of Medicine, Seoul, Korea; 2 Department of Psychiatry, Seoul National University College of Medicine, Seoul, Korea; 3 Kidney Research Institute, Seoul National University College of Medicine, Seoul, Korea; 4 Department of Internal Medicine, Seoul National University Bundang Hospital, Gyeonggi-do, Korea

## Abstract

**Background:**

Although health-related quality of life (HRQOL) is a potential independent predictor of mortality, nephrologists have shown little interest in HRQOL with respect to mortality in chronic kidney disease (CKD). The aim of this article is to evaluate the impact of HRQOL on mortality in the elderly, who are likely to develop or already have CKD.

**Methods:**

Among 1,000 randomly sampled participants aged more than 65 years (sourced from the Korean Longitudinal Study on Health and Ageing), 944 subjects were evaluated for HRQOL. HRQOL was assessed using a 36-item Short-Form health survey (SF36). A cumulative survival rate was calculated according to tertiles of SF36 scores and classified by the presence of CKD (estimated GFR <60 ml/min/1.73 m^2^).

**Results:**

Among 944 subjects, 46.6% had CKD. CKD patients had lower total and physical component scores compared with subjects without CKD. The 3-year cumulative survival rate was 90.0% (non-CKD vs. CKD: 92.6% vs. 87.4%, *P *= 0.005 by log rank test). After adjusting for multiple variables, a reduced SF36 score (physical and mental components) was a strong predictor of all-cause mortality. Physical components were consistently able to predict mortality after CKD classification, but mental components were statistically significant only in the CKD group.

**Conclusion:**

In addition to traditional risk factors of mortality, nephrologists should be aware of HRQOL as a predictor of mortality and should make efforts to improve HRQOL in CKD patients.

## Background

"The aging society" is a familiar term as elderly individuals (aged 65 years and older) make up an increasing proportion of the global population [1]. In line with global trends, the proportion of the elderly in Korea is increasing (8.3% in 2003) and is expected to reach 15% in 2019 [2]. This increase may lead to age-associated increases in chronic diseases. Chronic kidney disease (CKD) has been recognized as one of a number of chronic diseases primarily affecting the elderly. Worldwide, the prevalence of CKD in the elderly has increased to nearly half of the population aged 70 years and older [3], and the incidence among the elderly in Korea is similar [4]. Therefore, the problems associated with CKD need to be appropriately managed to reduce the burden of CKD in the aging society.

The ability to predict future morbidity and mortality is a key to reduce the burden of CKD. To this end, monitoring a patient's functional and subjective status of well-being, collectively known as health-related quality of life (HRQOL), is of particular importance in CKD patients [4-7]. Traditional risk factors (e.g., atherosclerosis, smoking, and diabetes mellitus) for mortality had been considered important in the elderly with or without CKD [8,9]; however, it is currently thought that traditional risk factors do not account for all reported mortality [10]. Recently, HRQOL, comprising physical, mental, and social health, is recognized as an important predictor of mortality in elderly individuals or patients with end-stage renal disease [5,7,11,12]. However, nephrologists have shown little interest in the role that HRQOL plays in mortality in CKD patients [13].

The 36-item Short-Form health survey (SF36) has been validated as a QOL assessment tool for a wide variety of patients, including CKD patients [14-16]. Here, we evaluate HRQOL using the SF36 and assess the impact of HRQOL on mortality in the elderly, who are likely to develop or already have CKD.

## Methods

### Study Participants

The present study was designed as a population-based, prospective cohort study of health, aging, and common geriatric diseases in a population aged ≥ 65 years in Seongnam-si, a satellite city of Seoul, Korea. The study design was described in detail elsewhere as an element of the Korean Longitudinal Study on Health and Aging (KLoSHA) [17]. The baseline phase of KLoSHA began in September 2005. The KLoSHA includes two cohorts, one selected from the total population of Seongnam-si (931,019 individuals) and a 6.6% sample of the population aged ≥ 65 years. For the elderly sample (Sample-RE), a simple random sample (1,118 individuals, 1.81%) was selected from a list of 61,730 residents aged ≥ 65 years as of August 1, 2005. The sampled subjects were invited to participate in the study by letter and telephone. Of the 1,118 candidates, 698 agreed to participate in the baseline KLoSHA study. For the "oldest old age" sample (Sample-OO), all individuals aged ≥ 85 years in Seongnam-si (3,136 persons) were contacted by letter and telephone, and 302 agreed to participate in the KLoSHA. We enrolled the Sample-OO in addition to the Sample-RE. All participants were Korean. From a random sample of 1,000 participants, we evaluated 944 who had examined the SF36. All assessments were performed at the Seoul National University Bundang Hospital in Gyeonggi-do, Korea. An independent ethics committee at each participating institution (SNUBH) approved the study protocol (B-0508/023-003). The study was conducted in accordance with the Declaration of Helsinki.

### Measurements and Definitions

The investigated clinical parameters included age, sex, ever-drinking, ever-smoking, and a history of hypertension, diabetes mellitus, coronary heart disease (CHD), or cerebrovascular accident (CVA). Systolic blood pressure (SBP) and diastolic blood pressure (DBP) were measured after participants had rested for at least 3 minutes. Numerous laboratory measurements were obtained. Serum measurements included hemoglobin, glucose, total protein, albumin, total cholesterol, triglyceride, high-density lipoprotein (HDL) cholesterol, and creatinine (Cr) levels. Urine measurements included a dipstick test for albumin and measurement of red blood cell (RBC) counts per high-power field by light microscopy. Glomerular filtration rate (GFR) was calculated for 932 participants using the Modification of Diet in Renal Disease (MDRD) Study equation [18].

Patients with one of the following were classed as hypertensive: SBP ≥ 140 mmHg, DBP ≥ 90 mmHg, or use of antihypertensive medication, irrespective of BP. Diabetes mellitus was defined as a fasting glucose level ≥ 126 mg/dL or the use of hypoglycemic agents. CHD was defined as self-reported history of angina pectoris, acute myocardial infarction, percutaneous coronary intervention, or coronary artery bypass operation. Proteinuria was defined as albumin ≥ 1+ and hematuria as an RBC count >5 per high-power field. CKD was defined as an estimated GFR <60 ml/min/1.73 m^2 ^[19].

The HRQOL of subjects was assessed using the Korean version of the SF36 [14,20]. It consists of 36 questions, 35 of which are included on eight multi-item scales: Physical Functioning, Role--Physical, Bodily Pain, General Health, Vitality, Social Functioning, Role--Emotional, and Mental Health. The SF36 assesses physical and mental health components. All-cause mortality data were obtained from the Ministry of Public Administration and Security's national database in June 2009.

### Statistical analysis

All analyses were performed using SPSS software (SPSS version 16.0, Chicago, IL, USA). Data were presented as mean/standard deviations (SD) for continuous variables and as proportions for categorical variables. Demographic and clinical data were described and compared between CKD and non-CKD groups. Differences were analyzed using the χ^2 ^test for categorical variables and the Student's *t *test for non-categorical variables. All subjects were divided into three groups according to tertiles of SF36 scores. The Kaplan-Meier method and the log rank test were used to assess and compare cumulative mortality rates between non-CKD and CKD groups. The unadjusted hazard ratios for all-cause mortality were calculated by the Cox proportional hazard model (model 1), and adjustments were made (model 2). Variables included for adjustments were age, gender, and others that had *P *values of less than 0.05 in univariate analyses. The hazard ratio for all-cause mortality was calculated after classifying subjects according to the presence of CKD. A *P *value of less than 0.05 was considered significant.

## Results

### Baseline characteristics of participants

Table 1 shows the patient baseline characteristics. Of the 944 participants, 424 (44.9%) were male and 520 (55.1%) were female. The mean age was 76 years (range, 65-98 years). The proportions with hypertension and diabetes were 71.1% and 20.9%, respectively. The mean creatinine level was 1.12 mg/dL and the mean GFR was 61.1 ml/min/1.73 m^2^. Among the participants, 46.6% had a GFR of less than 60 ml/min/1.73 m^2^; 8 (0.9%) had a GFR of 15-30 ml/min/1.73 m^2^, and 2 (0.2%) had a GFR of less than 15 ml/min/1.73 m^2^. The physical component score was significantly lower in CKD patients than in non-CKD subjects.

**Table 1:** Demographics of participants responding to the baseline Short Form with 36 questions

	CKD (-) (n = 488)	CKD (+) (n = 444)	Total subjects (n = 944)
Age^‡^	74.8/8.2	77.4/8.9	76.0/8.6
Male gender (%)^‡^	52.9	36.5	45.1
Hypertension (%) *	68.0	74.4	71.1
Diabetes mellitus (%)	20.9	20.5	20.9
CHD (%)^†^	4.7	10.6	7.6
CVA (%)	9.2	11.0	10.1
Ever-drinking (%)^‡^	45.5	32.4	39.2
Ever-smoking (%) *	43.1	35.7	39.6
SBP (mmHg)	132.6/17.8	132.2/18.3	132.5/18.1
DBP (mmHg)	82.8/10.2	82.5/11.0	82.7/10.6
Serum findings			
Hemoglobin (g/dL)^‡^	13.9/1.6	13.6/1.4	13.7/1.5
Glucose (mg/dL)^†^	111.0/28.0	106.4/20.9	108.9/25.1
Total protein (g/dL)	7.4/0.47	7.5/0.46	7.5/0.46
Albumin (g/dL)	4.09/0.27	4.09/0.22	4.09/0.24
Total cholesterol (mg/dL) *	199.5/37.5	205.7/37.6	202.7/37.9
Triglyceride (mg/dL)	131.1/88.8	137.8/72.9	135.0/82.1
HDL cholesterol (mg/dL)	60.8/16.0	59.5/14.8	60.2/15.4
Creatinine (mg/dL)^‡^	0.98/0.15	1.27/0.41	1.12/0.34
eGFR (ml/min/1.73 m^2^)^‡^	70.2/8.7	51.0/8.1	61.1/12.8
Proteinuria (%)	7.4	9.4	8.4
Hematuria (%)	9.9	11.3	10.8
SF36 score			
Physical functioning^†^	56.9/27.89	50.6/29.60	53.9/28.88
Role---physical^†^	71.1/28.46	65.3/32.70	68.4/30.67
Bodily pain	63.1/28.11	59.3/31.64	61.3/29.89
General health	43.8/21.85	42.7/22.05	43.3/21.94
Vitality	52.3/20.56	50.6/21.87	51.5/21.20
Social functioning	79.5/23.55	76.4/26.80	78.0/25.18
Role---emotional	81.4/26.00	78.3/29.36	79.9/27.68
Mental health	68.6/19.52	67.6/20.84	68.1/20.15
PCS^†^	55.8/13.3	53.1/14.6	54.5/14.0
MCS	52.2/10.1	51.1/11.0	51.7/10.5
Median follow-up (interquartile, months)	39 (35--42)	39 (35--41)	39 (35--42)

GFR was not estimated in twelve subjects.

P < 0.05, ^† ^P < 0.01, ^‡ ^P < 0.001

Abbreviations: CKD: chronic kidney disease; CHD: coronary heart disease; CVA: cerebrovascular accident; SBP: systolic blood pressure; DBP: diastolic blood pressure; HDL: high-density lipoprotein; eGFR: estimated glomerular filtration rate; SF36: 36-item Short-Form health survey; PCS: physical component summary score; MCS: mental component summary score.

During the 3-year follow-up period (0-45 months), 104 subjects (11.0%) died. Among the clinical and laboratory parameters in Table 1 (excluding the SF36 scores), the mortality rate was associated with age (52.3% older vs. 47.7% younger by a median age of 73 years: OR 5.97 (3.50-10.20), *P *< 0.001), ever-smoking (+39.6% vs. -60.4%: OR 1.60 (1.08-2.35), *P *= 0.018), proteinuria (+8.4% vs. -91.6%: OR 2.08 (1.20-3.61), *P *= 0.009), serum levels of hemoglobin (1 g/dL increase: OR 0.83 (0.74-0.94), *P *= 0.002), total protein (1 g/dL increase: OR 0.50 (0.33-0.75), *P *= 0.001), albumin (1 g/dL increase: OR 0.17 (0.10-0.27), *P *< 0.001), total cholesterol (1 mg/dL increase: OR 0.99 (0.99-1.00), *P *= 0.004), HDL cholesterol (1 mg/dL increase: OR 0.98 (0.97-1.00), *P *= 0.007), and estimated GFR (CKD vs. non-CKD: OR 1.74 (1.17-2.58), *P *= 0.006). These variables were used for adjustments in multivariate analyses. The 3-year cumulative survival rate was 90.0% (non-CKD group vs. CKD group: 92.6% vs. 87.4%, *P *= 0.005 by the log rank test).

### Influence of health-related quality of life on all-cause mortality

Each 10-unit increase on the eight scales was associated with decreased all-cause mortality in study participants after adjustment for multiple variables (Table 2). Univariate analysis showed that groups with high scores of SF36 components (except general health) had greater survival rates than groups with low scores. This trend remained consistent after adjustment (model 2).

**Table 2:** Hazard ratios for all-cause mortality according to SF36 scores (n = 944)

	Model 1^a^		Model 2^b^	
	
SF36 survey	HR (95% CI)	*P *value	HR (95% CI)	*P *value
10-unit increase in SF36 components				
Physical functioning	0.78 (0.72--0.83)	<0.001	0.81 (0.75--0.89)	<0.001
Role---physical	0.85 (0.81--0.90)	<0.001	0.90 (0.85--0.96)	0.001
Bodily pain	0.90 (0.85--0.96)	0.002	0.92 (0.86--0.99)	0.020
General health	0.93 (0.85--1.02)	0.123	0.90 (0.82--1.00)	0.052
Vitality	0.85 (0.77--0.93)	<0.001	0.88 (0.79--0.98)	0.018
Social functioning	0.83 (0.78--0.90)	<0.001	0.87 (0.81--0.93)	<0.001
Role---emotional	0.84 (0.80--0.89)	<0.001	0.88 (0.83--0.93)	<0.001
Mental health	0.85 (0.78--0.93)	0.001	0.83 (0.75--0.91)	<0.001
Physical component summary score				
Tertile 1 (<49.4)	1 (Reference)		1 (Reference)	
Tertile 2 (49.4--61.7)	0.46 (0.30--0.72)	0.001	0.54 (0.33--0.87)	0.011
Tertile 3 (>61.7)	0.25 (0.14--0.43)	<0.001	0.35 (0.19--0.64)	0.001
Mental component summary score				
Tertile 1 (<49.0)	1 (Reference)		1 (Reference)	
Tertile 2 (49.0--57.0)	0.68 (0.44--1.05)	0.082	0.63 (0.40--1.01)	0.055
Tertile 3 (>57.0)	0.36 (0.21--0.61)	<0.001	0.39 (0.22--0.70)	0.001

^a ^Unadjusted model.

^b^Model adjusted for age, gender, ever-smoking, proteinuria, serum levels of hemoglobin, total protein, albumin, cholesterol, HDL cholesterol, and estimated GFR.

Abbreviations: SF36: 36-item Short-Form health survey; HR: hazard ratio; CI: confidence interval.

Upper tertiles in physical and mental component scores were more highly correlated with improved survival rate than lower tertiles. Using 10-unit increases in physical and mental components, the increase in each score was associated with reduced mortality: the physical component score had an adjusted OR of 0.71 ((0.61-0.84), *P *< 0.001); the mental component score had an adjusted OR of 0.65 ((0.54-0.78), *P *< 0.001).

**Figure 1:**
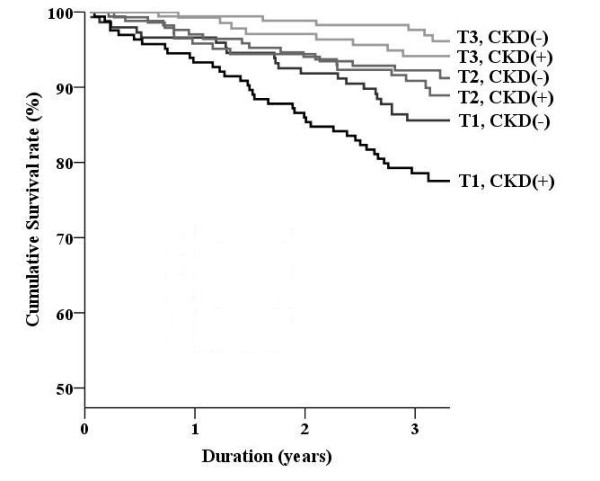
**Survival curves classified by the presence of chronic kidney disease: physical component score**. The Kaplan-Meier curve is assessed in participants according to the physical component score. Tertiles in physical component score: T1, <49.4; T2, 49.4--61.7; T3, >61.7.

**Figure 2:**
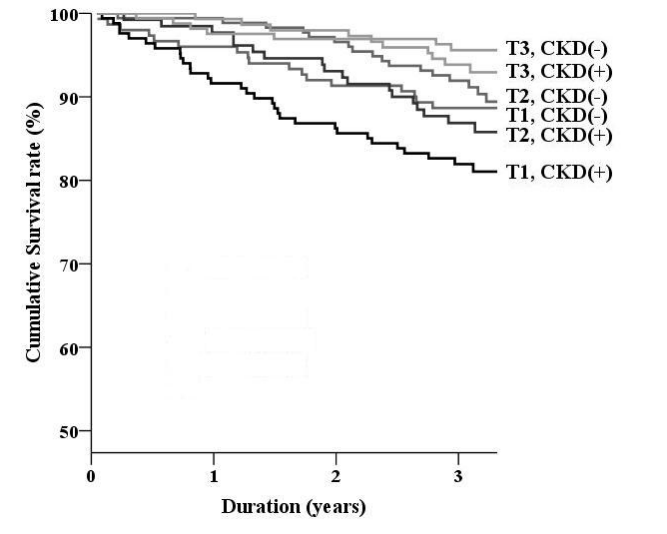
**Survival curves classified by the presence of chronic kidney disease: mental component score**. The Kaplan-Meier curve is assessed in participants according to the mental component score. Tertiles in mental component score: T1, <49.0; T2, 49.0--57.0; T3, >57.0.

We divided subjects into two groups on the basis of presence of CKD. Figure 1 and Figure 2 show the Kaplan-Meier curve according to tertiles of SF36 scores and the presence of CKD. Cumulative survival rates according to physical and mental component scores were further separated by the presence of CKD (*P *< 0.001 in Figure 1 and Figure 2 by the log rank test). By multivariate analysis (Table 3), the physical component score was associated with all-cause mortality, irrespective of CKD. The mental component score was significantly correlated with all-cause mortality in the CKD group; however, the correlation was only marginally significant in the non-CKD group.

**Table 3:** Stratified analysis of all-cause mortality by the presence of chronic kidney disease

	CKD (-) (n = 488)		CKD (+) (n = 444)	
	
SF36 survey	HR (95% CI)	*P *value	HR (95% CI)	*P *value
Physical component summary score				
Tertile 1 (<49.4)	1 (Reference)		1 (Reference)	
Tertile 2 (49.4--61.7)	0.44 (0.21--0.92)	0.029	0.54 (0.28--1.05)	0.068
Tertile 3 (>61.7)	0.23 (0.09--0.60)	0.003	0.44 (0.20--0.98)	0.045
Mental component summary score				
Tertile 1 (<49.0)	1 (Reference)		1 (Reference)	
Tertile 2 (49.0--57.0)	0.67 (0.33--1.37)	0.273	0.51 (0.27--0.99)	0.041
Tertile 3 (>57.0)	0.41 (0.16--1.05)	0.051	0.35 (0.17--0.74)	0.006

Model adjusted for age, gender, ever-smoking, proteinuria, serum levels of hemoglobin, total protein, albumin, cholesterol, HDL cholesterol, and estimated GFR.

Abbreviations: CKD, chronic kidney disease; SF36, 36-item Short-Form health survey; HR, hazard ratio; CI, confidence interval.

## Discussion

Clinicians focus on newer risk factors (e.g., inflammation, oxidative stress, and epigenetic change) as well as traditional risk factors (e.g., atherosclerosis, smoking, and diabetes mellitus) when assessing mortality risk in patients with CKD [21]. It is essential to evaluate non-typical risk factors because the aging society is more heavily confronted with various non-traditional co-morbidities. In the present study, we evaluated the relationship between HRQOL, as measured by the SF36, and all-cause mortality in an elderly population with or without CKD. Both physical and mental components, including each health component, affected all-cause mortality in elderly subjects. This trend was consistent for individuals with CKD. To the best of our knowledge, although HRQOL is known to be related to mortality in patients with end-stage renal disease, no report has been published on the correlation between HRQOL and mortality in patients with CKD. This issue should be kept in mind by nephrologists who may otherwise not be interested in the QOL of CKD patients.

The SF36 is an approved test that is applicable to healthy elderly individuals as well as CKD patients [14-16]. Han et al. also used the SF36 for assessing HRQOL in elderly Koreans (n = 219, aged 73.7 years) [22]. The SF36 scores for each component were similar between Han's cohort and our study cohort, but scores of social functioning, role emotional, and mental health were higher in our cohort. Recently, Mujais et al. reported HRQOL in CKD patients using the Kidney Disease Quality of Life (KDQOL) questionnaire, which combines the generic SF36 instrument with a kidney disease-specific instrument [23]. In their study, the mean physical and mental component scores for stage 3 CKD patients were 40 and 51, respectively. Although comparison between two cohorts is impossible because of different baseline characteristics (low proportion of diabetes mellitus, high serum levels of hemoglobin and albumin in our cohort), we assumed that SF36 scores from the randomly sampled design of our cohort could be representative of HRQOL among the elderly in Seongnam-si.

The association between negative self-assessment of health status and mortality in the elderly has been noted for more than 30 years, since Maddox's report [24]. Bernard et al. evaluated the relationship between self-assessment of health status and the subsequent risk of death among the elderly during a 2.5-year period [12]. When 3,485 elderly subjects were asked, "How would you rate your health at the present time?", the group with an answer of "poor" (the most negative of five possible responses) had an adjusted hazard ratio of 5.5 (4.0-7.5) for mortality compared with those who answered "excellent" (the most positive of five possible responses). Several hypotheses have been proposed to explain the correlation between HRQOL and clinical outcome. For example, a negative self-evaluation of health could stimulate a psychoneuroimmunologic response, reflect the elderly person's accurate self-detection of preclinical changes in body function, or lead to a delay in taking health-protective and maintenance actions [12,25]. In some reports, HRQOL has been shown to be related to nutritional factors, such as cholesterol and hemoglobin levels [4,5]. However, it is not fully understood why subjective assessment of health status is strongly correlated with objective clinical outcome after adjustment for chemical markers. Interestingly, the mental component score did not differ between two groups that were divided by an eGFR of 60 ml/min/1.73 m^2^. However, the mental component score in the CKD group was a greater predictor of mortality than in the non-CKD group. The reason for the weak correlation between the mental component score and mortality in the elderly without CKD is not clear. It might be because sample number was modest after classification or that elderly individuals without CKD were more strongly affected by the physical component than by the mental component. On the other hand, some studies have reported that a depressed mental state is common and clearly correlated with mortality in patients with end-stage renal disease, consistent with our results [26,27].

The importance of HRQOL in patients with end-stage renal disease is well known. DeOreo et al. examined the SF36 score in 1,000 patients who underwent hemodialysis [28]; the median values of the physical and mental component scores were 37 and 47, respectively. It was revealed that a low SF36 score, particularly a low physical component score, was associated with increased hospitalization and mortality rates. Kalantar-Zadeh et al. also measured SF36 scores in 65 end-stage renal disease patients and found a correlation with hospitalization and mortality; the mental component score and total score had the strongest correlations [5]. Furthermore, these researchers revealed that serum albumin and hemoglobin levels were associated with SF36 scores. However, few studies have investigated CKD patients with reduced renal function (GFR less than 60 ml/min/1.73 m^2^). The present study showed that elderly CKD patients had lower HRQOL scores than subjects without CKD; this reduced HRQOL score was strongly associated with increased all-cause mortality after adjustment for traditional risk factors and chemical markers. Furthermore, as shown in Figure 1 and Figure 2, HRQOL further separated the mortality rate according to the presence of CKD. In this regard, we should treat HRQOL and CKD equally.

Although our results are informative, our study is not without limitations. First, because all participants in our cohort were elderly Asian individuals, our results may not be applicable to other ethnic groups or adults aged <65 years with CKD. Indeed, Lopes et al. have reported differences in HRQOL among various ethnic groups undergoing hemodialysis [29]. Second, the MDRD equation has not yet been investigated in the Korean population and may not be appropriate for the elderly population. Third, HRQOL was measured only once at the start of the present prospective research; therefore, our data do not show changes during the follow-up period. Fourth, HRQOL was higher than expected (mean value: 54.5 in the physical component; 51.7 in the mental component). However, the purpose of our study was not to evaluate the cut-off point of the SF36 score. Rather, we sought to understand an independent correlation between HRQOL and mortality in CKD patients.

## Conclusion

The present study demonstrated that HRQOL was a strong predictor of all-cause mortality in an elderly Asian population, including subjects with CKD. Although the present study does not reveal the mechanism by which the subjective HRQOL is correlated with the objective outcome of mortality, it would be reasonable to try to improve HRQOL in the elderly and CKD patients to reduce mortality. This is a responsibility of clinicians that will be more important in the aging society as the prevalence of CKD increases. Further studies are needed to ascertain how interventions to increase HRQOL can reduce mortality rates in CKD patients.

## Competing interests

The authors declare that they have no competing interests.

## Authors' contributions

**SSH **participated in the design of the study and performed the statistical analysis. **SSH **also wrote this paper. **KWK **participated in the design of the study. **KYN **collected data and performed the statistical analysis. **DWC **helped to draft the manuscript. **YSK **collected data. **SK **participated in the design of the study. **HJC **participated in the design of the study and finally approved this paper to be submitted. All authors read and approved this paper.

## Pre-publication history

The pre-publication history for this paper can be accessed here:

http://www.biomedcentral.com/1471-2369/10/39/prepub
